# Constructing Pairing-Friendly Elliptic Curves under Embedding Degree 1 for Securing Critical Infrastructures

**DOI:** 10.1371/journal.pone.0161857

**Published:** 2016-08-26

**Authors:** Maocai Wang, Guangming Dai, Kim-Kwang Raymond Choo, Prem Prakash Jayaraman, Rajiv Ranjan

**Affiliations:** 1 School of Computer, China University of Geosciences, Wuhan, Hubei, China; 2 Hubei Key Laboratory of Intelligent Geo-Information Processing, China University of Geosciences, Wuhan, Hubei, China; 3 Department of Information Systems and Cyber Security, University of Texas at San Antonio, San Antonio, Texas, United States of America; 4 RMIT University, Melbourne, Australia; 5 University of Newcastle, Newcastle, United Kingdom; Nankai University, CHINA

## Abstract

Information confidentiality is an essential requirement for cyber security in critical infrastructure. Identity-based cryptography, an increasingly popular branch of cryptography, is widely used to protect the information confidentiality in the critical infrastructure sector due to the ability to directly compute the user’s public key based on the user’s identity. However, computational requirements complicate the practical application of Identity-based cryptography. In order to improve the efficiency of identity-based cryptography, this paper presents an effective method to construct pairing-friendly elliptic curves with low hamming weight 4 under embedding degree 1. Based on the analysis of the Complex Multiplication(CM) method, the soundness of our method to calculate the characteristic of the finite field is proved. And then, three relative algorithms to construct pairing-friendly elliptic curve are put forward. 10 elliptic curves with low hamming weight 4 under 160 bits are presented to demonstrate the utility of our approach. Finally, the evaluation also indicates that it is more efficient to compute Tate pairing with our curves, than that of Bertoni et al.

## 1 Introduction

Many countrieshave thrived on the wealth fromthe information technologies(IT) have enabled, and IT forms the backbone of many aspects of the critical infrastructure sectors [1, 2]. There are 16 critical infrastructure sectors in the U.S. [3]. As noted by both scholars [4–10] and government agencies, such as U.S. Homeland Security, the critical infrastructure represents systems and assets, and it is also defined in detailed [3].

The interconnective of the systems in the critical infrastructure sector, and the increasing sophistication, scale and the persistent nature of cyber attacks against such systems, can potentially result in equipment being forced to operate beyond its intended design and safety limits, resulting in cascading system malfunctions and shut downs such as the collapse of an entire electricity grid; or operating procedures or conditions being manipulated to slow the effort of restoring essential services [11, 12]. It is, therefore, unsurprising that the cyber security of a nation’s critical infrastructure (including assets, networks, and systems) is regarded as a top priority of national security by countries around the world [13–17].

One of the key requirements in critical infrastructure cyber security is information confidentiality, and the cryptography is generally the core technology to provide information confidentiality [18].

Identity-based cryptography(IBC) is a relatively new branch of cryptography, which can directly compute a user’s public key using publicly available information from the user’s identity [19]. Therefore, one does not need to distribute his digital certificate signed by a certificate authority (CA), or query the certificate database to get the other party’s public key when conducting electronic transactions. In other words, IBC resolves the challenges and complexity associated with certificate management and traditional public-key cryptosystem. A limitation of IBC is, however, the computation cost involving in constructing the pairings [20]. IBC has the subject of various research, but it remains a topic of ongoing research interest, and one of the research challenges is the generation of efficient parameters such as pairing-friendly elliptic curves.

The existing efficient algorithms to compute Weil and Tate pairings [21, 22] are generally based on Miller’s algorithm [23]on (hyper) elliptic curves. One line of research which focuses on reducing the loop in Miller’s algorithm was initiated by Duursma-Lee [24] and, subsequently extended by Barreto et al. [25] to supersingular abelian varieties.

In practice, the cryptographic pairings used to construct these systems are based on the Weil and Tate pairings on elliptic curves over finite fields [26]. Both pairings are a bilinear map from an elliptic curve group on the finite field *F*_*p*_ to the multiplicative group of some extension field $F_{p^{k}}$. The parameter *k* is called the embedding degree of the elliptic curve. The pairing is considered to be secure if both discrete logarithms in the groups *E*(*F*_*p*_) and $F_{p^{k}}$ are computationally infeasible.

To optimize the application performance, the parameters *p* and *k* should be determined according to this standard that both discrete logarithm problems approximately have the equal difficulty when using the best known algorithms. Moreover, a large prime factor *r* should be included in the order of the group #*E*(*F*_*p*_). For example, if the large prime factor *r* ≥ 2^160^, the pairing is generally considered to be safe against existing attacks. Therefore, it is essential to be able to construct elliptic curves efficiently for arbitrary *p* and *k* values to differ the security level or to meet the requirement of discrete log in future improvements. This is the gap we attempt to address in this paper.

This paper is organized as follows. In the next two sections, we introduce the reader to related literature and Tate pairing, respectively. In Section 4, we describe our approach to constructing pairing-friendly elliptic curves under embedding degree 1 and preliminary evaluation results to demonstrate utility and practicality. Our discussion and concluding remarks are provided in the last two sections.

## 2 Related Work

Constructing elliptic curves with various embedding degrees has been the subject of ongoing research. For example, Cocks and Pinch [27] constructed the curves with arbitrary embedding degree *k*, but the efficiency is very low because the size *q* of the field *F*_*p*_ is limited by the subgroup of prime order r with *q* ≈ *r*^2^. Fotiadis and Konstantinou [28] presented two general methods to produce sparse families and applied them to four embedding degrees *k*, where *k*. Barreto and Naehrig [29] constructed the curves of prime order with *k* = 12. Freeman [30] proposed a construction for the curves with embedding degree *k* = 10. A complete characterization of common elliptic curves of prime order with *k* = 3, 4, or 6, is provided by Miyaji, Nakabayashi, and Takano [31]. Menezes, Okamoto, and Vanstone [32] illustrated that embedding degree *k* should be not more 6 in a supersingular elliptic curve, especially *k* ≤ 3 and *k* ≠ 2 or *k* ≠ 3. Some researches [33] reduced the ratio $p=\frac{\text{log }p}{\text{log }r}$ for arbitrary *k* between the characteristicp of the finite field and the prime order *r* of the subgroup. However, no concrete examples have been proposed with *ρ* small enough to construct curves with prime order.

In fact, if *k* = 1, the pairing will become a bilinear map from the elliptic curve group on the finite field *F*_*p*_ to the elliptic curve group on the same finite field *F*_*p*_. In other words, we would not involve the extension field $F_{p}^{k}$ when computing the pairing, which is the constraintin pairing-based cryptography applications.

Izuta, Nogami and Morikawa [34] proposed a method for generating a certain composite order ordinary pairing-friendly elliptic curve of embedding degree 1. In their method, the order has two large prime factors such as the modulus of RSA cryptography. Lee and Park [35] proposed a new algorithm to construct Brezing-Weng-like elliptic curves having the Complex Multiplication(CM) equation of degree 1, as well as presenting new families of curves with larger discriminants.

It is clear from the literature that pairing-friendly elliptic curves under embedding degree 1 are constructed on the base field, rather than the extension field, which can significantly improve the computation efficiency of Tate pairing. This is the gap that this paper attempts to address. More specifically, this paper proposes an effective method to construct pairing-friendly elliptic curves with low hamming weight 4 under embedding degree 1.

## 3 Tate Pairing

In practice, as the theoretical model is unknown, we use the Monte-Carlo method [36] to generate the required data based on a fixed theoretical model.

Weil pairing was first introduced into cryptography by Menezes, which was used to study the elliptic curve discrete logarithm problem on certain elliptic curves [32]. Extending on the work of Menezes, Frey introduced Tate pairing to cryptography [37], which is now widely used to design pairing-based cryptosystems because Tate pairing is twice as efficient as Weil pairing.

Let E be an elliptic curve over a finite field *F*_*p*_, and *r* be a positive integer which is co prime to *p*. In most applications, *r* is a prime and *r*|#*E*(*F*_*p*_). Let *k* be a positive integer such that the field $F_{p^{k}}$. contains the r-th roots of unity, and *k* is called the embedding degree. Then Tate pairing is a mapping [38]:

$$t\left(P,Q\right):E\left(F_{p^{k}}\right)\left[r\right]\times E\left(F_{p^{k}}\right)/rE\left(F_{p^{k}}\right)\to F_{p^{k}}^{*}/{\left(F_{p^{k}}^{*}\right)}^{r}$$

According to the definition of Tate pairing, if the embedding degree *k* ≠ 1, then the computation of Tate pairing is related to the extension field $F_{p^{k}}$, and the computation process will be time-consuming. However, if the embedding degree *k* = 1, the computation of Tate pairing only runs on the base field $F_{p^{k}}$ rather than the extension field $F_{p^{k}}$. This will greatly improve the computation efficiency of Tate pairing.

In Tate pairing, both the point *P* and the point *Q* are from two different subgroups with the same order r as subgroup $E(F_{p^{k}}[r])$ and $(F_{p^{k}}^{*}{)}^{r}$ respectively. That is to say, if *k* = 1, then the point *P* and the point *Q* come from two different subgroup *G*_1_ and *G*_2_ of *E*(*F*_*p*_) with the same order *r*, and *G*_1_ ∩ *G*_2_ = ∅. However, “How to construct the elliptic curve which includes two different groups with the same order *r* when *r* is a large prime with *r* ≥ 2^160^?” and “How to find the two different groups?” are two key challenges in designing pairing-friendly elliptic curves under embedding degree 1.

In this paper, we propose an effective algorithm to construct pairing-friendly elliptic curves under embedding degree 1. In our algorithm, it can be ensured that both the point *P* and the point *Q* are from two different subgroups with the same order *r*, which enables the computation of Tate pairing to run only on the base field.

## 4 Constructing Pairing-friendly Elliptic Curves

In this section, a new method to generate pairing-friendly elliptic curves is proposed, which comprises three algorithms as follows.

1. The first algorithm is used to generate a large prime of low hamming with weight 4.
2. The second algorithm is used to generate the finite field *p*, the order *u* of a non-supersingular elliptic curve over *F*_*p*_, the order *r* of a point on the elliptic curve.
3. The last algorithm is used to construct pairing-friendly elliptic curves under embedding degree 1.

### 4.1 The Construction Method

In the common method [31, 35]to construct elliptic curves, the equation *u* = *p* + 1 ± *W* is used to generate the parameters of the elliptic curves. This equation provides a means to determine the order #*E* of an elliptic curve *E* according to the characteristic p of the finite field *F*_*p*_. However, the order #*E* generated using the equation is generally unable to meet the security requirement. Therefore, it is a challenge to generate a suitable elliptic curve using the common method. Moreover, even if a suitable elliptic curve can be generated, it will take a long time. For example, in the method of Izuta, Nogami and Morikawa [34], it will take about 20 hours to generate an elliptic curve.

In our method, we present a new equation *p* = *u* ± *W* + 1 to generate the parameters of elliptic curves. On first glance, the new equation may appear similar to the common equation. However, in the new equation, the order #*E* is known, and we need to obtain *p* from the order #*E* (rather than the order #*E* from p). Thus, we only need to determine the characteristic *p* of the finite field *F*_*p*_ from the order #*E* of an elliptic curve *E*, and our algorithm 2 describes the process required to generate *p* from the order #*E*. In other words, we can generate an elliptic curve under arbitrary order, while the order #*E* of an elliptic curves *E* can be trivially obtained using *u* = *r* * *r* (from the security requirement), where *r* is a large prime, and *r* has a low hamming with weight 4 (based on our algorithm 1). As the order of the subgroup is a large prime of low hamming with weight 4, the efficiency of generating elliptic curves is significantly improved. More specifically, our method requires about 200 ms to generating a suitable elliptic curve.

**Theorem 1** If *E is a non-super singular elliptic curve over F*_*p*_ *with order u, D is the* CM discriminant for *p*, according to the discriminant condition 4*p* = *W*^2^ + *DV*^2^ and *u* = *p* + 1 ± 1, then

p=u±X+1

where *W* = *X* ± 2, *V* = *Y*.

Proof. It is well known that the CM discriminant *D* for *p* meets the Eqs (1) and (2) for every non-super singular elliptic curve over *F*_*p*_ with order *u*.

$$4p=W^{2}+DV^{2}$$ (1)

u=p+1±W (2)

The Eq (3) can be gotten from the Eq (2).

4u=4p+4±4W (3)

The Eq (4) can be gotten by replacing 4*p* with the Eq (1) in the Eq (3).

$$4u=W^{2}+DV^{2}+4\pm 4W$$ (4)

The Eq (4) can be written as the Eq (5).

$$4u={\left(W\pm 2\right)}^{2}+DV^{2}$$ (5)

The Eq (5) can be written as the Eq (6).

$$4u=X^{2}+DY^{2}$$ (6)

where *X* = *W* ± 2 and *Y* = *V*.

Therefore, the Eq (1) can be converted to the Eq (7) with *X* = *W* ± 2.

$$4p={\left(X\pm 2\right)}^{2}+DY^{2}$$ (7)

The Eq (8) can be gotten from the Eqs (6) and (7)

4p=4u±4X=4 (8)

The Eq (8) can be be written as the Eq (9)

p=u±X+1 (9)

This ends the proof.

Theorem 1 provides a method to calculate the characteristic *p* of the finite field *F*_*p*_ according to the order *u* of an elliptic curve. That is to say, for any elliptic curve with the order *u* expected, we can easily calculate the characteristic *p* of the finite field *F*_*p*_ according to the Eq (9). This is a new way, which can generate an elliptic curve under any order we expected.

In Miller algorithm of computing Tate pairing, if some bit of the binary representation for the order r of subgroup is ‘1’, operators would be needed to compute multiplication and inverse operations [39]. Otherwise, (i.e. if the binary bit is ‘0’), no additional operator is needed. It is clear that the process to compute Tate pairing will be more efficient if the binary representation of the order r has fewer ‘1’ bits and more ‘0’ bits. This forms the basis of the three relative algorithms.

### 4.2 Algorithm 1

Algorithm 1 outlines the method to generate a large prime of low hamming with weight 4. In other words, there are only two ‘1’ bits in addition to the highest bit and the lowest bit in the binary representation for the large prime. The large prime will be used as the order *r* of subgroup in algorithms 2 and 3.

In algorithm 1, the input parameter is the length *m*(*m* ≥ 160) of the binary representation for the large prime, the output result is the large prime r of low hamming with weight 4.

Algorithm 1. Generating a large prime of low hamming with weight 4.

Input: The length *m*(*m* ≥ 160) of the binary representation for the large prime; a positive integer *t* for the number of trials.

Output: The large prime *r* of low hamming with weight 4.

step 1 Choose random *s*, *t* in the interval (0, *m* − 1) to ensure 0 < *s* < *t* < *m* − 1;
step 2 *r* ← 2^0^ + 2^*s*^ + 2^*t*^ + 2^*m* − 1^;
step 3 Compute *v* and an odd value *w*, such that *r* − 1 = 2^*v*^ *w*
step 4 For *j* from 1 to *t* do
  step 4.1 Choose random *a* in the interval 0 < *a* < *r*;
  step 4.2 Set *b* ← *a*^*w*^ mod *r*
  step 4.3 If *b* = 1 or *b* = *r* − 1, goto step 4.6;
  step 4.4 For *i* from 1 to *v* − 1 do
    step 4.4.1 Set *b* ← *b*^2^ mod *r*
    step 4.4.2 If *b* = *r* − 1 goto step 4.6;
    step 4.4.3 if *b* = 1, goto step 1;
    step 4.4.4 Next *i*.
  step 4.5 goto step 1;
  step 4.6 Next *j*;
step 5 Output *r*.

### 4.3 Algorithm 2

Algorithm 2 describes the method to generate the finite field *p*, the order u of a non-supersingular elliptic curve over *F*_*p*_, and the order *r* of a point on the elliptic curve according to the length *m*(*m* ≥ 160) of the finite field *p*.

Algorithm 2. Generating the finite field *p*, the order *u* of a non-supersingular elliptic curve over *F*_*p*_, and the order *r* of a point on the elliptic curve.

Input: The length *m*(*m* ≥ 160) of the finite field *p*.

Output: The finite field *p*, the order *u* of a non-super singular elliptic curve over *F*_*p*_, the order *r* of a point on the elliptic curve.

step 1 Generate a large prime *r* of low hamming with weight 4 using algorithm 1;
step 2 Compute the order u of a non-supersingular elliptic curve *u* = *r*^2^;
step 3 Assign *D* = 3, set *X* = *r*, *Y* = *r*, such that the values of both *X* and *Y* satisfy the condition 4*u* = *X*^2^ + *DY*^2^;
step 4 Compute *p* = *r*^2^ + *r* + 1 according to *p* = *u* ± *X* + 1 when *u* = *r*^2^, *X* = *r*;
step 5 If *p* is not a prime, goto Step 1;
step 6 Output the finite field *p*, the order *u* of a non-supersingular elliptic curve over *F*_*p*_, the order *r* of a point on a elliptic curve.

We would also remark that “the IEEE Standard Specifications for Public-Key Cryptography” [40] recommends that in the construction of a curve with prescribed CM, if *D* = 3, the coefficients *a*_0_ and *b*_0_ of *E* should be 0 and 1 respectively.

### 4.4 Algorithm 3

Algorithm 3 presents the method to construct pairing-friendly elliptic curves under embedding degree 1. We assume that there are two different subgroups with the same order *r* on the elliptic curve generated by algorithm 3, where *r* is a large prime.

In algorithm 3, the input parameter is the length *m*(*m* ≥ 160) for the subgroup order, and the output results are *a*, *b* and the prime *p* as the parameters of the elliptic curve *y*^2^ ≡ *x*^3^ + *ax* + *b* mod *p*, low hamming prime *r* as the order of subgroup, point *P*_1_ as the base point for generating subgroup *G*_1_ while calculating Tate pairing, where *rP*_1_ = 0, and point *P*_2_ as the base point for generating subgroup *G*_2_ while calculating Tate pairing where *rP*_1_ = 0, *rP*_2_ = 0 and *G*_1_ ∩ *G*_2_ = ∅.

Algorithm 3 is designed to be convenient for users generating pairing-friendly elliptic curves under embedding degree 1, as the only input parameter is the length of the binary representation for the order *r* of the subgroup. Algorithm 3 runs by calling algorithm 2, which in turn calls algorithm 1.

Algorithm 3. Constructing pairing-friendly elliptic curves.

Input: The length *m*(*m* ≥ 160) for the subgroup order.

Output: *a*, *b* and the prime *p* denote the parameters of the elliptic curve *y*^2^ ≡ *x*^3^ + *ax* + *b* mod *p*, low hamming order *r* denotes the order of subgroup, point *P*_1_(*rP*_1_ = 0) and point *P*_2_(*rP*_2_ = 0).

step 1 Generate the finite field *p*, the order *u* of a non-supersingular elliptic curve over *F*_*p*_, the order *r* of a point on the elliptic curve using algorithm 2;
step 2 Select an integer *ζ* with 0 < *ζ* < *p*;
step 3 Set *a* ← 0 and *b* ← *b*_0_ζ mod *p*;
step 4 Locate a point *P*_1_ with order *r* on the curve *y*^2^ ≡ *x*^3^ + *ax* + *b* mod *p*.
step 5 If the output of Step 4 is in the wrong order, goto Step 2.
step 6 Locate a point *P*_2_ with order r on the curve *y*^2^ ≡ *x*^3^ + *ax* + *b* mod *p*, where *P*_2_ ∉ {*kP*_1_|*k* ∈ {1, 2…, *r*}}.
step 7 The output *p*, *a*, *b* as the parameters of the elliptic curve *y*^2^ ≡ *x*^3^ + *ax* + *b* mod *p*, the large prime *r* with low hamming weight as the order of subgroup, the point *P*_1_ as the base point for generating subgroup *G*_1_ while calculating Tate pairing, where *rP*_1_ = 0, and the point *P*_2_ as the base point for generating subgroup *G*_2_, while *rP*_2_ = 0 and *G*_1_ ∩ *G*_2_ = ∅.

The elliptic curve generated by algorithm 3 can potentially include two different subgroups *G*_1_ and *G*_2_, with large prime order *r* with low hamming weight for computing Tate pairing. Because the order *r* of subgroup is a public parameter, these parameters generated by the algorithms presented in the paper do not impact on the security of Pairing-based cryptosystems(PBC).

### 4.5 Preliminary Findings

We implement the construction described in Section 4.1 using Pentium 4 PC (CPU 3.06GHz), and the findings are as follows.

Algorithm 1:

*r* = 730750818686719107034401070324602422792720220161 = 2159 + 2124 + 228 + 20

Algorithm 2:

*p* = 53399675901131022287481940452568268554861807611629722703698801201 2286237103997609758897031086083

*r* = 730750818686719107034401070324602422792720220161

*u* = *r*^2^ = 53399675901131022287481940452568268554861807611556647621830129 2905251836033673007336104310865921

Algorithm 3:

Table 1 describes 10 elliptic curves generated by algorithm 3 under the above *p*, *r*, *u*.

**Table 1:** 10 pairing-friendly curves with low hamming weight 4 under given *p*, *r*, *u*(*r* with 160 bits).

Parameters	b	*P*_1_	*P*_2_
The 1^st^ group	5582	(92216901,324171638614811955738700451351453938462743355913304125667979195175749628071873615636670182400781)	(2900911840,470565327089465608766717290556724343611875827253253971039274229090627830093258979017255917746829)
The 2^nd^ group	411	(6456,200783653312643253000427685185361672824889578029877838466376294032581210908578307946592006236274)	(7718449758221,246594700063950682499345743858581550409793007029702423620631032309187268322219527336805985703980)
The 3^rd^ group	6888558	(63,483447298606802197007667782086007062212874881060089832042154800397367248862591909523693105053579)	(503,77335678175724311469109067552943334864235153595640796029200821503606472539285202409938146494560)
The 4^th^ group	1852511737533	(28136114,242158699775814654792914165109030127513135019773154553510244588647142304534134418272078033955490)	(86590,121551762235427048604306704938720769730830601113589433595621413717086224050169417598237928322315)
The 5^th^ group	111158	(9069952,191884782129835076896430782729279489410474467097317768476761535512656282281715989056396995028939)	(40,362063866070142646287391901715038404328540944674222712928339139412213864564948793769019222805261)
The 6^th^ group	7134	(352,373120637403567989697297819791130549970377887442049625481567602466046997349176087351878502769389)	(1171827216,497218853134580777525964760410326161237125053509830407941623317513914592421492486740293952308203)
The 7^th^ group	562	(7751170,170089631638765123245772026413070351656555635734890000712753208456308661954335842959630945562639)	(9123978,491696588250751128316690742989376247219892973435085382978639044870863996676582978948360971631311)
The 8^th^ group	1105557501121	(96209917051711,525449385155426365101090731761951180537095809586740545805755386839131992568764934554139827350205)	(12835,74127495296715015118497558174623723156473745792295184182056487700402725864604342423009775785761)
The 9^th^ group	814	(76110676327,455230770668674635001073148949612110022716252695998918206294806414038955424454903144922555015137)	(856171875,173617915786825757928929966543011205848033233611185463361858494144333951770711174509831733937961)
The 10^th^ group	1110977	(935542646001,425605723028492010195922479602517755514636860155519341225132168354176911010027713634301519279864)	(211058810,424408607333334853102700723328774094337451473846030924917501392093461859795155755424956700868152)

## 5 Discussion

In the Miller algorithm, for every bit of the order *r* of the subgroup, we would need to compute 16 multiplication and 7 inverse operations. If the bit is 1, however,we would need to compute 11 multiplication and five inverse operations. For the order r of the subgroup with 160 bits in ordinary PBCs, there are 80 ‘1’ bits on average. Therefore, we would need to compute 3,429 multiplication and 1,515 inverse operations. It is pleasing to note that using the parameters in our approach, we only need 2,593 multiplication and 1,135 inverse operations, as shown in Table 2.

**Table 2:** Efficiency analysis.

	The ordinary PBC				PBC with parameter in the paper			
	Every bit (160 bits)		Every bit with 1 (79 bits)		Every bit (160 bits)		Every bit with 1 (3 bits)	
	Multiple	Inverse	Multiple	Inverse	Multiple	Inverse	Multiple	Inverse
	16	7	11	5	16	7	11	5
	2560	1120	869	395	2560	1120	33	15
Total	Multiple:3429 Inverse:1515				Multiple:2593 Inverse:1135			

An inverse operation is estimated to be 5.18 multiplication operations [39], and implementing our method outlined in this paper will save 24.9% of the time required to compute the Tate pairing:

$$\frac{2593+1135*5.18}{3429+1515*5.18}=0.751=75.1\%$$

To demonstrate the practicality of the new method we proposed,using the parameters with 160 bits presented in Table 1, we implement a proof-of-concepton a Pentium 4 PC (CPU 3.06GHz) in Table 3, using the parameters with 160 bits presented in Table 1.

**Table 3:** Comparative summary of Tate pairing computations.

Parameters	The result from Bertoni et al. [39]	The result from this paper
Platform	PentiumIII @ 1GHz	Pentium IV@ 3.06GHz
Length of prime	160 bits	160 bits
Low Hamming Weight	3	4
Time for a Tate pairing	41ms	12.93ms

**Fig 1:**
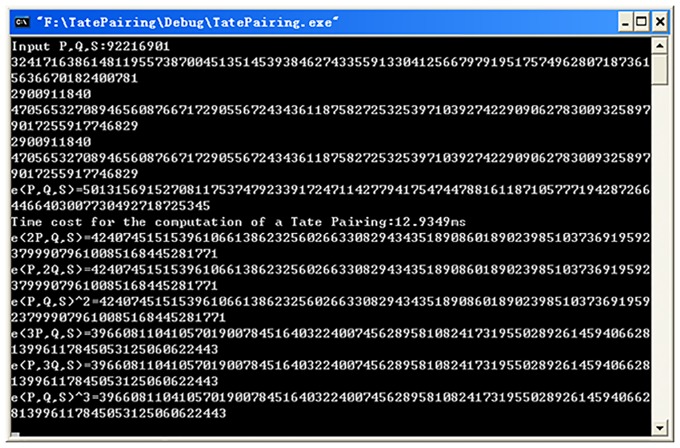
The result of computing Tate pairing on the first group curve. The first 9 lines gives the parameters of the first group curves. Then the result of *e*(*P*, *Q*), *e*(2*P*, *Q*), *e*(*P*, 2*Q*), *e*(3*P*, *Q*), *e*(*P*, 3*Q*), *e*(*P*, *Q*)^2^ and *e*(*P*, *Q*)^3^ are given and the bilinear property is verified.

As shown in Fig 1., our implementation takes 12.93 ms to compute a pairing. We then compared with the findings from Bertoni et al. [39], as shown in Table 3. In the latter, the large prime of the order of the subgroup is 160 bits, but with a Hamming weight equal to 3 and the embedding degree of 2. As shown in Table 3, our algorithm is more computationally efficient compared to that of Bertoni et al.

The computation results depicted in Fig 1. can also be verified using the bilinear characteristic of Tate pairing, as explained below:

$$t\left(P,2Q\right)=t\left(2P,Q\right)=t{\left(P,Q\right)}^{2}$$

$$t\left(P,3Q\right)=t\left(3P,Q\right)=t{\left(P,Q\right)}^{3}$$

Recall that in Tate pairing, if the embedding degree *k* ≠ 1, then the computation of Tate pairing is related to the extension field $F_{p^{k}}$, which is very time consuming. Building on Miller’s algorithm, we present an effective algorithm to construct pairing friendly elliptic curves with low hamming weight 4 under embedding degree 1, which enables the computation of Tate pairing only on the base field.

## 6 Conclusion

Ensuring information confidentiality in critical infrastructures will be increasingly important in our increasingly interconnected world. In this paper, we studied the generation method of pairing-friendly elliptic curves for identity-based cryptography(IBC), with the aim to significantly improve the computation efficiency of IBC. We demonstrated how pairing-friendly elliptic curves can be efficiently conducted, both in theory and practice which can be deployed in critical infrastructure systems, such as cyber-physical systems with limited resources [40]. In our approach,pairings computing requires only the base field, rather than the extension field.

More specifically, in this paper, we described and conducted a preliminary analysis of the new method to construct pairing-friendly elliptic curves under embedding degree 1. Unlike the existed traditional CM methods,the parameters are not randomly generated in our method. The parameters are computed under a given expression, which significantly improves the efficiency of generating elliptic curve. Moreover, in our algorithm, the only input parameter is the binary length of the large prime *r*, and then all parameters of the elliptic curve can be rapidly generated. Our method consists of three algorithms, namely: an algorithm to generate low hamming prime *r* according to the expected length of the large primer, which is also used as the order of the subgroup; an algorithm to calculate the character *p* of the finite field *F*_*p*_ and the order *u* of the elliptic curve according to the prime *r*; and an algorithm to generate the pairing-friendly elliptic curves and the two different points *P*_1_ and *P*_2_ on the elliptic curve with the same order *r*. It also ensures *G*_1_ ∩ *G*_2_ = ∅, where *G*_1_ is the subgroup generated by *P*_1_ and *G*_2_ is the subgroup generated by *P*_2_, *G*_1_ and *G*_2_ are two different subgroups of *E* with the same order *r*.

The paper also provided 10 elliptic curves with low hamming, weight 4 under 160 bits generated using our algorithms, which demonstrated the utility of our method. Then, we demonstrated the practicality of our method by implementing the method using Tate pairing.

Our curves can be applied in real word such as Internet of Things(IoT), Electronic Commerce(EC) and Copyright Protection(CP). In fact, in all fields, which are involved in public key cryptography, the proposed method can be applied to implement digital signature, key management and authentication protocol [41–43]. The future work includes two aspects. The first aspect is to optimize Miller’s algorithm to improve the computation efficiency of Tate pairing. The other aspect is to apply the elliptic curves constructed by our method to the practical cryptosystem.

## Supporting Information

S1 File Pairing-friendly elliptic curves under embedding degree 1 with 160 bits. There are 10 group pairing-friendly elliptic curves under embedding degree 1 with 160 bits. In every group, the parameters of *p*, *r*, #*E*, *b*, *P*, *Q* are given. The parameters of *a* is equal 0 in all groups.
(PDF)

S2 File Pairing-friendly elliptic curves under embedding degree 1 with 190 bits. There are 10 group pairing-friendly elliptic curves under embedding degree 1 with 160 bits. In every group, the parameters of *p*, *r*, #*E*, *b*, *P*, *Q* are given. The parameters *a* is equal 0 in all groups.
(PDF)

## References

[1] Dong MX, Ota K, Laurence T, et al LSCD: A Low-Storage Clone Detection Protocol for Cyber-Physical Systems. IEEE Transactions on Computer-Aided Design of Integrated Circuits and Systems, 2016, 35(5):712–723. 10.1109/TCAD.2016.2539327

[2] Wang L., Tao J., Ranjan R., et al G-Hadoop: MapReduce across distributed data centers for data-intensive computing. Future Generation Computer Systems, 2013, 29(3):739–750 10.1016/j.future.2012.09.001

[3] Kepler D, Heasley P Implementation of EO 13636 and PPD-21: Draft Report and Recommendations. National Infrastructure Advisory Council, 2013

[4] Zhao J., Wang L., Tao J., etc. A security framework in G-Hadoop for big data computing across distributed Cloud data centres. Journal of Computer and System Sciences, 2014, 80(5):994–1007. 10.1016/j.jcss.2014.02.006

[5] Chen D., Liu Z., Wang L.,etc. On-demand service hosting on production grid infrastructures. MONET, 2013, 18(5): 651–663.

[6] Wang L., Kurze T., Tao J., etc. Natural Disaster Monitoring with Wireless Sensor Networks: A Case Study of Data-intensive Applications upon Low-Cost Scalable Systems. The Journal of Supercomputing, 2013, 66(3): 1178–1193.

[7] Wang L., Fu C. Research Advances in Modern Cyberinfrastructure. Generation Comput, 2010, 28(2): 111–112. 10.1007/s00354-009-0077-9

[8] Zhang W., Wang L., Liu D., etc. Towards building a multi-datacenter infrastructure for massive remote sensing image processing. Concurrency and Computation: Practice and Experience, 2013, 25(12): 1798–1812. 10.1002/cpe.2966

[9] Wang L., Chen D., Hu Y., etc. Towards enabling Cyberinfrastructure as a Service in Clouds. Computers & Electrical Engineering, 2013, 39(1): 3–14. 10.1016/j.compeleceng.2012.05.001

[10] Raymond C A conceptual interdisciplinary plug-and-play cyber securityframework In Kaur H & Tao X, editors, ICTs and the Millennium Development Goals A United Nations Perspective, pp. 81–99, New York, USA: Springer, 2014.

[11] Raymond Choo K. The cyber threat landscape: Challenges and future research directions. Computers & Security, 2011, 30(8): 719–731. 10.1016/j.cose.2011.08.004

[12] Eisentrager K, Lauter K, Montgomery P. Fast elliptic curve arithmetic and improved Weil pairing evaluation. Proc. of the 2003 RSA conference on The cryptographers’ track, Berlin, Germany, pp. 343–354, 2003.

[13] Raymond Choo K. High tech criminal threats to the national information infrastructure. Information Security Technology Report, 2010, 15(3): 104–111. 10.1016/j.istr.2009.09.001

[14] Cornish P, Livingstone D, Clemente D, et al. Cyber Security and the UK’s Critical National Infrastructure. A Chatham House Report, 2011.

[15] Yang Y, Lu J, Raymond C, et al. On Lightweight Security Enforcement in Cyber-physical Systems. In Proceedings of International Workshop on Lightweight Cryptography for Security & Privacy (LightSec 2015), Bochum, Germany, Lecture Notes in Computer Science, Springer-Verlag, 2015.

[16] Dong M, Ota K, Laurence T, et al LSCD: A Low-Storage Clone Detection Protocol for Cyber-Physical Systems. IEEE Transactions on Computer-Aided Design of Integrated Circuits and Systems, 2016, 35(5):712–723. 10.1109/TCAD.2016.2539327

[17] Dong M, Li H, Ota K, et al Rule caching in SDN-enabled mobile access networks. IEEE Network, 2015, 29(4):40–45. 10.1109/MNET.2015.7166189

[18] Mao W. Modern Cryptography: Theory and Practice. Prentice Hall, 2003

[19] Joye M., Neven G. Identity-based Cryptography. IOS Press, 2008

[20] Moody D., Peralta R., Perlner R., etc. Report on Pairing-based Cryptography. Journal of Research of the National Institue of Standards and Technology, 2015, 120:11–27. 10.6028/jres.120.002 PMC4730686 26958435

[21] Rahuman A, Athisha G. Reconfigurable Architecture for Elliptic Curve Cryptography Using FPGA. Mathematical Problems in Engineering, 2013, 2013:1–8. 10.1155/2013/675161

[22] Dai G, Wang M, Peng L, et al Implementation and Optimization for Tate pairing. Intelligent automation and soft computing, 2011, 17(5):607–617. 10.1080/10798587.2011.10643174

[23] Miller V. The Weil pairing and its efficient calculation. Journal of Cryptology, 2004, 17(4):235–261. 10.1007/s00145-004-0315-8

[24] Duursma I, Lee H S Tate Pairing Implementation for Hyperelliptic Curves *y*^2^ = *x*^*p*^ − *x* + *d*. Proc. of Advances in Cryptology-Asiacrypt, 2003, Heibelberg,Germany, pp. 111–123.

[25] Barreto P, Galbraith S, Eigeartaigh C, et al Efficient pairing computation on supersingular abelian varieties. Designs, Codes and Cryptography, 2007, 42(3): 239–271. 10.1007/s10623-006-9033-6

[26] Barreto P, Galbraith S, Eigeartaigh C, et al An efficient key-policy attribute-based encryption scheme with constant ciphertext length. Mathematical Problems in Engineering, 2013, 2013:1–7.

[27] Cocks C, Pinch R. Identity-based cryptosystems based on the Weil pairing. unpublished manuscript, 2001.

[28] Fotiadis G, Konstantinou E. More Sparse Families of Pairing-Friendly Elliptic Curves. Proc. Proc. of 13th International Conference on Cryptology and Network Security, Heraklion, Greece, pp.384–399, 2014.

[29] Barreto P, Naehrig M. Pairing-friendly elliptic curves of prime order. Proc. Of Selected Areas in Cryptography—12th International Workshop, Kingston, Canada, pp. 319–331, 2005.

[30] Freeman D Constructing Pairing-Friendly Elliptic Curves with Embedding Degree 10. Proc. Of algorithmic number theory, Berlin, Germany, pp.452–465, 2006.

[31] Miyaji A, Nakabayashi M, Takano S. New explicit conditions of elliptic curve traces for FR-reduction. IEICE Transactions on Fundamentals, 2001, vol. E84-A, no.5, pp.1234–1243.

[32] Menezes A, Okamoto T, Vanstone S. Reducing elliptic curve logarithms to logarithms in a finite field. IEEE Transactions on Information Theory, 1993, 39(5): 1639–1646. 10.1109/18.259647

[33] Brezing F, Weng A Elliptic curves suitable for pairing based cryptography. Designs, Codes, and Cryptography, 2005, 37(1):133–141. 10.1007/s10623-004-3808-4

[34] Izuta T, Nogami Y, Morikawa Y. Ordinary Pairing Friendly Curve of Embedding Degree 1 Whose Order Has Two Large Prime Factors. Proc. of IEEE Region 10 Conference on TENCON 2010, Fukuoka, JAPAN, pp.769–772, 2010.

[35] Lee H, Park C. Generating Pairing-Friendly Curves with the CM Equation of Degree 1. Proc. of 3rd International Conference on Pairing-Based Cryptography, Palo Alto, California, USA, pp.66–77, 2009.

[36] Pollard J. A monte carlo method for factorization. BIT Numerical Mathematics, 1975, 15(3): 331–334. 10.1007/BF01933667

[37] Frey G and Ruck H. A remark concerning m-divisibility and the discrete logarithm in the divisor class group of curves. Mathematics of Computation, 1994, 62(206): 865–874. 10.2307/2153546

[38] Zhang F, Naini R and Susilo W. An efficient signature scheme from bilinear pairings and its application. Proc. Of Advances in Cryptography-PKC’04, Heidelberg, Germany, pp. 277–290, 2004.

[39] Bertoni G, Chen L, Fragneto P, et al. Computing Tate Pairing on Smartcards. Proc. Of Proceedings of Workshop on Cryptographic Hardware and Embedded Systems, Edinburgh, Scotland, 2005.

[40] IEEE-SA Standards Board. IEEE standard specifications for public-key cryptography, 2000.

[41] Perera C, Ranjan R, Wang L, et al Big Data Privacy in the Internet of Things Era. IT Professional, 2015, 17(3): 32–39. 10.1109/MITP.2015.34

[42] Kolodziej J, Khan S, Wang L, et al Security, energy, and performance-aware resource allocation mechanisms for computational grids. Future Generation Computer Systems, 2014(31):77–92.

[43] Wei J, Cai W, Wang L, et al A secure information service for monitoring large scale gridse. Parallel Computing, 2007, 33(7–8): 572–591.

