# Supplementary material for: Constructing Pairing-Friendly Elliptic Curves under Embedding Degree 1 for Securing Critical Infrastructures
Source: PLoS One. 2016 Aug 26;11(8):e0161857. doi: 10.1371/journal.pone.0161857 (PMC5001717; doi:10.1371/journal.pone.0161857)
Supplement: S1 File — There are 10 group pairing-friendly elliptic curves under embedding degree 1 with 160 bits. In every group, the parameters of p, r, #E, b, P, Q are given. The parameters of a is equal 0 in all groups. (PDF) [file pone.0161857.s001.pdf]

Pairing-friendly elliptic curves under embedding degree 1 with 160 bits

The 1st Group:
P:5339967590113102228748194045256826855486180761162972270369880120122862371039
97609758897031086083
R:730750818686719107034401070324602422792720220161
#E:533996759011310222874819404525682685548618076115566476218301292905251836033
673007336104310865921
B:5582
BasePoint1:
X=92216901
Y=3241716386148119557387004513514539384627433559133041256679791951757496280718
73615636670182400781
BasePoint2:
X=2900911840
Y=4705653270894656087667172905567243436118758272532539710392742290906278300932
58979017255917746829

The 2nd Group:
P:5339967590113102228748194045256826855486180761162972270369880120122862371039
97609758897031086083
R:730750818686719107034401070324602422792720220161
#E:533996759011310222874819404525682685548618076115566476218301292905251836033
673007336104310865921
B:411
BasePoint1:
X=6456
Y=2007836533126432530004276851853616728248895780298778384663762940325812109085
78307946592006236274
BasePoint2:
X=7718449758221
Y=2465947000639506824993457438585815504097930070297024236206310323091872683222
19527336805985703980

The 3rd Group:
P:5339967590113102228748194045256826855486180761162972270369880120122862371039
97609758897031086083
R:730750818686719107034401070324602422792720220161
#E:533996759011310222874819404525682685548618076115566476218301292905251836033
673007336104310865921
B:6888558
BasePoint1:
X=63

Y=483447298606802197007667782086007062212874881060089832042154800397367248862591909523693105053579
BasePoint2:
X=503
Y=773356781757243114691090675529433348642351535956407960292008215036064725392852024099381464945 60

The 4th Group:
P:533996759011310222874819404525682685548618076116297227036988012012286237103997609758897031086083
R:730750818686719107034401070324602422792720220161
#E:533996759011310222874819404525682685548618076115566476218301292905251836033673007336104310865921
B:1852511737533
BasePoint1:
X=28136114
Y=242158699775814654792914165109030127513135019773154553510244588647142304534134418272078033955490
BasePoint2:
X=86590
Y=121551762235427048604306704938720769730830601113589433595621413717086224050169417598237928322315

The 5th Group:
P:533996759011310222874819404525682685548618076116297227036988012012286237103997609758897031086083
R:730750818686719107034401070324602422792720220161
#E:533996759011310222874819404525682685548618076115566476218301292905251836033673007336104310865921
B:111158
BasePoint1:
X=9069952
Y=191884782129835076896430782729279489410474467097317768476761535512656282281715989056396995028939
BasePoint2:
X=40
Y=362063866070142646287391901715038404328540944674222712928339139412213864564948793769019222805261

The 6th Group:
P:533996759011310222874819404525682685548618076116297227036988012012286237103997609758897031086083
R:730750818686719107034401070324602422792720220161
#E:533996759011310222874819404525682685548618076115566476218301292905251836033

673007336104310865921
B:7134
BasePoint1:
X=352
Y=373120637403567989697297819791130549970377887442049625481567602466046997349176087351878502769389
BasePoint2:
X=1171827216
Y=497218853134580777525964760410326161237125053509830407941623317513914592421492486740293952308203

The 7th Group:
P:533996759011310222874819404525682685548618076116297227036988012012286237103997609758897031086083
R:730750818686719107034401070324602422792720220161
#E:533996759011310222874819404525682685548618076115566476218301292905251836033673007336104310865921
B:562
BasePoint1:
X=7751170
Y=170089631638765123245772026413070351656555635734890000712753208456308661954335842959630945562639
BasePoint2:
X=9123978
Y=491696588250751128316690742989376247219892973435085382978639044870863996676582978948360971631311

The 8th Group:
P:533996759011310222874819404525682685548618076116297227036988012012286237103997609758897031086083
R:730750818686719107034401070324602422792720220161
#E:533996759011310222874819404525682685548618076115566476218301292905251836033673007336104310865921
B:1105557501121
BasePoint1:
X=96209917051711
Y=525449385155426365101090731761951180537095809586740545805755386839131992568764934554139827350205
BasePoint2:
X=12835
Y=74127495296715015118497558174623723156473745792295184182056487700402725864604342423009775785761

The 9th Group:

P:533996759011310222874819404525682685548618076116297227036988012012286237103997609758897031086083
R:7307508186867191070344010703246024227927202201 61
#E:5339967590113102228748194045256826855486180761155664762183012929052518360336730073361043108659 21
B:814
BasePoint1:
X=76110676327
Y=455230770668674635001073148949612110022716252695998918206294806414038955424454903144922555015137
BasePoint2:
X=856171875
Y=173617915786825757928929966543011205848033233611185463361858494144333951770711174509831733937961

The 10th Group:
P:533996759011310222874819404525682685548618076116297227036988012012286237103997609758897031086083
R:730750818686719107034401070324602422792720220161
#E:533996759011310222874819404525682685548618076115566476218301292905251836033673007336104310865921
B:1110977
BasePoint1:
X=935542646001
Y=425605723028492010195922479602517755514636860155519341225132168354176911010027713634301519279864
BasePoint2:
X=211058810
Y=424408607333334853102700723328774094337451473846030924917501392093461859795155755424956700868152
