# Supplementary material for: Constructing Pairing-Friendly Elliptic Curves under Embedding Degree 1 for Securing Critical Infrastructures
Source: PLoS One. 2016 Aug 26;11(8):e0161857. doi: 10.1371/journal.pone.0161857 (PMC5001717; doi:10.1371/journal.pone.0161857)
Supplement: S2 File — There are 10 group pairing-friendly elliptic curves under embedding degree 1 with 160 bits. In every group, the parameters of p, r, #E, b, P, Q are given. The parameters a is equal 0 in all groups. (PDF) [file pone.0161857.s002.pdf]

Pairing-friendly elliptic curves under embedding degree 1 with 190 bits

The 1st Group:
P:1539140867046659344229650023804789370652393001467846500621008774065822235748
89954918829075571057020921478142492673
R:392318858461667547739736841485780351462856018272408567809
#E:1539140867046659344229650023804789370652393001467846500624931962650438911226
29691760314855922519876939750551060481
B:19939371
BasePoint1:
X=101632576
Y=6456559677953595243249071440914812438670419830497516231661845169016266961252
740373819864329712585532393724507088
BasePoint2:
X=787
Y=6220736531753030307986847442468196865829099988742248512394484480366664588240
3064689325461240631945228940714376579

The 2nd Group:
P:1539140867046659344229650023804789370652393001467846500621008774065822235748
89954918829075571057020921478142492673
R:392318858461667547739736841485780351462856018272408567809
#E:1539140867046659344229650023804789370652393001467846500624931962650438911226
29691760314855922519876939750551060481
B:567184
BasePoint1:
X=1092
Y=5033293955454477678469984701810665151705107883585503907273070179125238546293
8704508376635308546678472360680194081
BasePoint2:
X=121367159060718627217224114111511210822
Y=5062495179398117315482452195131861205687573535018516000473775772685569826079
755771680166540725218316793325840517

The 3rd Group：
P:1539140867046659344229650023804789370652393001467846500621008774065822235748
89954918829075571057020921478142492673
R:392318858461667547739736841485780351462856018272408567809
#E:1539140867046659344229650023804789370652393001467846500624931962650438911226
29691760314855922519876939750551060481
B:2814077581
BasePoint1:
X=19690
Y=1370181361676906110049947576963562847982320575824315330887337760475050712678

036598686744623133561998748646 6881155

B:55
BasePoint1:
X=7
Y=40781893368993891930812730536689867310229831329456416865097402286437497676197476657785564193107114768744579183989
BasePoint2:
X=92078397
Y=38327133129293669369124895928441182276403452219975048333880879711264470590128625938318345399691475052346563594639

The 7th Group：
P:153914086704665934422965002380478937065239300146784650062100877406582223574889954918829075571057020921478142492673
R:392318858461667547739736841485780351462856018272408567809
#E:153914086704665934422965002380478937065239300146784650062493196265043891122629691760314855922519876939750551060481
B:210268
BasePoint1:
X=3
Y=25259660275166042476508412818690894830751911798540833801742046007436348972333155985743154873490688791912437370362
BasePoint2:
X=9383
Y=52818386380563806820207122278047300826779124594300805906198741479519251647974475752603835500891253269097507643048

The 8th Group：
P:153914086704665934422965002380478937065239300146784650062100877406582223574889954918829075571057020921478142492673
R:392318858461667547739736841485780351462856018272408567809
#E:153914086704665934422965002380478937065239300146784650062493196265043891122629691760314855922519876939750551060481
B:3355
BasePoint1:
X=4
Y=33465854102021863879037655179003797550899359455298861446808538532759621833106544313173837553567863238959501414853
BasePoint2:
X=935066
Y=25060571103401030699045863598927025276604971750063955392610457560402236138031328696567467112995035667549312514645

The 9th Group：
P:1539140867046659344229650023804789370652393001467846500621008774065822235748

899549188290755710570209214781424926 73
R:392318858461667547739736841485780351462856018272408567809
#E:15391408670466593442296500238047893706523930014678465006249319626504389112 2
62969176031485592251987693975055106048 1
B:51
BasePoint1:
X=50346
Y=57555532573640818827262748847267684541640923059596625583351763446875768566 06
38701938804298512772145565858141594 09
BasePoint2:
X=28814
Y=28032432633508388655760174858705934639900674679722533643331232902816297271 95
01059475107515893562331302673619867 09

The 10th Group：
P:15391408670466593442296500238047893706523930014678465006210087740658222357 48
899549188290755710570209214781424926 73
R:392318858461667547739736841485780351462856018272408567809
#E:15391408670466593442296500238047893706523930014678465006249319626504389112 2
62969176031485592251987693975055106048 1
B:16185
BasePoint1:
X=878931311568
Y=99490923932604876235586753114779739238539443160377567427764146713645715091 18
36342886413585157257505583452878562 0
BasePoint2:
X=1501877
Y=25570786605891227931846965514724775085110343083163901243286674938445026013 07
24666343951399047250293087198096304 76
